# Role of Muscarinic Acetylcholine Signaling in Gastrointestinal Cancers

**DOI:** 10.3390/biomedicines7030058

**Published:** 2019-08-10

**Authors:** Mitsuru Konishi, Yoku Hayakawa, Kazuhiko Koike

**Affiliations:** Department of Gastroenterology, Graduate School of Medicine, The University of Tokyo, Tokyo 1138655, Japan

**Keywords:** muscarinic acetylcholine receptor, tuft cell, gastric cancer, colon cancer

## Abstract

In the tumor microenvironment, various stromal and immune cells accumulate and interact with cancer cells to contribute to tumor progression. Among stromal players, nerves have recently been recognized as key regulators of tumor growth. More neurotransmitters, such as catecholamines and acetylcholine (ACh), are present in tumors, as the cells that secrete neurotransmitters accumulate by the release of neurotrophic factors from cancer cells. In this short review, we focus on the role of nerve signaling in gastrointestinal (GI) cancers. Given that muscarinic acetylcholine receptor signaling seems to be a dominant regulator of GI stem cells and cancers, we review the function and mechanism of the muscarinic ACh pathway as a regulator of GI cancer progression. Accumulating evidence suggests that ACh, which is secreted from nerves and tuft cells, stimulates GI epithelial stem cells and contributes to cancer progression via muscarinic receptors.

## 1. Interaction between Nerves and Cancers

There has been accumulating evidence on the neural regulation of tissue stem cells and the promoting role of nerves and neurotransmitters in cancer initiation and progression [1,2,3,4,5,6,7,8,9]. For instance, Brownell et al. reported that a nerve-dependent microenvironment creates a molecularly and phenotypically distinct population of hair follicle stem cells via the Sonic hedgehog (SHH) pathway [1]. Hanoun et al. reported that the sympathetic nervous system promotes the infiltration of leukemic cells into bone marrow in an *MLL-AF9* AML model [2]. Katayama et al. reported that hematopoietic stem and progenitor cells in the bone marrow are regulated by the sympathetic nervous system, which attracts hematopoietic stem cells to their niche [3]. Magnon et al. reported that the autonomic nerve fibers, both adrenergic and cholinergic, in the prostate gland regulate prostate cancer development and dissemination in mouse models [4]. The authors later revealed that during this process, adrenergic signals regulate angiogenesis in prostate cancer tissues via metabolic changes in endothelial cells [10,11]. Mendez-Ferrer et al. reported that Nestin-expressing mesenchymal stem cells (MSCs) constitute an essential hematopoietic stem cell niche via adrenergic receptor signaling [5]. Peterson et al. reported that *Gli1*-expressing progenitors within mechanosensory touch dome epithelia are activated by sensory nerves via Hedgehog signaling in normal touch domes and contribute to tumorigenesis, while denervation attenuates these tumors [6]. Stopczynski et al. reported that neurotrophic factors and sensory nerves are increased during pancreatic cancer development, and metastatic tumor cells could be found along with the celiac and sensory ganglia in the spinal cord [7]. Furthermore, Renz et al. demonstrated that adrenergic signals promote pancreatic cancer progression, while cholinergic signals inhibit pancreatic cancer progression [12,13]. Venkatesh et al. reported that the synaptic protein neuroligin-3 (NLGN3) activates the PI3K-mTOR pathway and induces the feedforward expression of *NLGN3* in glioma cells, and NLGN3 promotes robust high-grade glioma cell proliferation [8]. Zhao et al. reported that the vagus nerve contributes to gastric tumorigenesis via muscarinic acetylcholine receptor 3-mediated Wnt signaling in the gastric stem cells [9]. Thus, nerves and neuronal signals widely regulate cancer progression within the tumor microenvironment.

Neurite outgrowth from nerves is caused by the neurotrophic factors secreted from cancer cells. On the other hand, nerves, which can be adrenergic, cholinergic, or of sensory origin, release various neurotransmitters from their endings and stimulate stromal cells, immune cells, and cancer cells, resulting in the promotion of cancer initiation, progression, and metastasis [14]. Therefore, there is a bidirectional interaction between nerves, cancer cells, and other stromal cells, and nerve signaling plays a central role in the complex tumor microenvironment.

## 2. Muscarinic Acetylcholine (ACh) Receptors

Muscarinic ACh receptors are members of the G protein-coupled receptors (GPCRs) that activate 5′-phosphate (G) proteins. GPCRs regulate a variety of biological processes by modulating the activation of adenylyl cyclase, the turnover of phosphatidylinositol lipid, and the status of ion channels [15,16,17]. Like other GPCRs, muscarinic ACh receptors have seven transmembrane helical domains that are connected by three extracellular and three intracellular loops. To date, five muscarinic ACh receptor subtypes have been identified and termed as CHRM1–5. These receptors regulate various cellular functions through different signaling pathways [17,18,19]. In general, CHRM1, CHRM3, and CHRM5 activate the phospholipid turnover and change the cellular calcium concentration. CHRM2 and CHRM4 inhibit the activity of adenylyl cyclase and reduce levels of cyclic adenosine monophosphate (cAMP). Muscarinic ACh receptors are widely expressed throughout the body, including in the gut, brain, eye, heart, vasculature, etc. [20,21,22,23,24,25,26,27,28]. In particular, CHRM1 and CHRM3 are robustly expressed in the gastrointestinal (GI) tract. In the stomach, ACh is secreted from vagus nerve endings, and gastric acid secretion from parietal cells is caused by CHRM3 [29,30,31,32], while pepsinogen secretion from gastric chief cells is promoted by CHRM1 and CHRM3 [33,34,35,36]. In addition, a low expression of CHRM5 has been reported in the gastric epithelium [9,37]. CHRM1 and CHRM3 appear to be expressed in normal colonic epithelia as well [17,38]. The influence of muscarinic receptors is highly diversified, depending on the cellular environment and proteome [39].

## 3. ACh Signaling and Gastric Cancer

In a recent study involving three mouse models of gastric cancer (genetically engineered, chemically induced, and *Helicobacter pylori*-induced), surgical vagotomy significantly attenuated gastric tumorigenesis. In addition, pharmacological denervation using botulinum toxin A or a blockade of muscarinic acetylcholine receptor 3 (M3R) inhibited tumorigenesis [9,40]. Interestingly, Lgr5+ gastric antral stem cells highly express *Chrm3*, the gene encoding M3R, suggesting that Lgr5+ stem cell function is modulated by M3R signaling [9]. Indeed, denervation inhibits Wnt signaling in gastric stem cells and suppresses stem cell expansion during carcinogenesis. In humans, tumor stage is correlated with nerve density in gastric cancer tissues, whereas vagotomy reduces the risk of gastric cancer recurrence [9]. 

**Figure 1:**
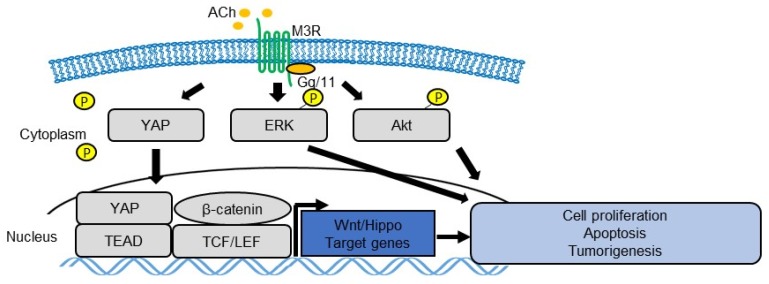
Schema of ACh-muscarinic acetylcholine receptor 3 (M3R) signaling pathway in gastrointestinal (GI) tumor cells. ACh-M3R signaling regulates cell proliferation, survival, and tumorigenesis by activating various signaling pathways, such as mitogen-activated protein kinase (MAPK), Akt, yes-associated protein (YAP), and Wnt.

Like other GPCRs, the activation of M3R leads to a variety of biochemical and electrophysiological responses, but the resulting physiological effects depend on the cell type and/or tissue type. M3R has been suggested to activate mitogen-activated protein kinase (MAPK) [41,42,43,44], Akt [25,43,45], or ras homolog family member A (RhoA) [46,47], thereby contributing to tumor growth in various cancers [48] (Figure 1). Subsequent studies have shown that M3R signaling promotes cellular proliferation and inhibits apoptosis via the epidermal growth factor receptor (EGFR) and Akt pathways in human gastric cancer cell lines and xenografts [49,50]. 

The yes-associated protein (YAP), a downstream effector of the Hippo pathway, regulates various cellular functions, such as proliferation, survival, stemness, and pluripotency [51,52]. In the Apc-dependent gastrointestinal tumors, YAP is highly upregulated and activated, and YAP activation appears to be required for the growth of Apc/β-catenin-dependent tumors [53,54,55]. Indeed, YAP regulates tissue regeneration and tumorigenesis in multiple organs, including the stomach and intestines, by activating tissue stem cells [56,57,58]. It was also suggested that types of GPCRs that activate G12/13, Gq/11, or Gi/o can activate YAP by suppressing its phosphorylation, whereas types of GPCRs that mainly activate Gs signaling can phosphorylate and, therefore, inhibit the YAP pathway [59]. Zhou et al. reported that SRY-Box9 (SOX9), which is known to be upregulated in gastric tissues during carcinogenesis [60], regulates the expression and phosphorylation of YAP in gastric cancer cell lines [61]. They also reported that SOX9 enhanced the proliferation, invasion, and migration of gastric cancer cells, and SOX9 may enhance the epithelial–mesenchymal transition (EMT) in gastric cancer cells via the Hippo-YAP pathway [61], supporting the notion that the Hippo-YAP pathway plays a critical role in gastric cancer progression. Similarly, our group demonstrated that M3R activates YAP signaling in gastric cancer cells, similar to the actions of other Gq/11 family receptors, and regulates the subsequent activation of Wnt in gastric tumors [48]. Thus, M3R and YAP could be a potential therapeutic target of nerve-dependent cancer development [51,62] (Figure 1). 

## 4. Tuft Cells as a Source of ACh

**Figure 2:**
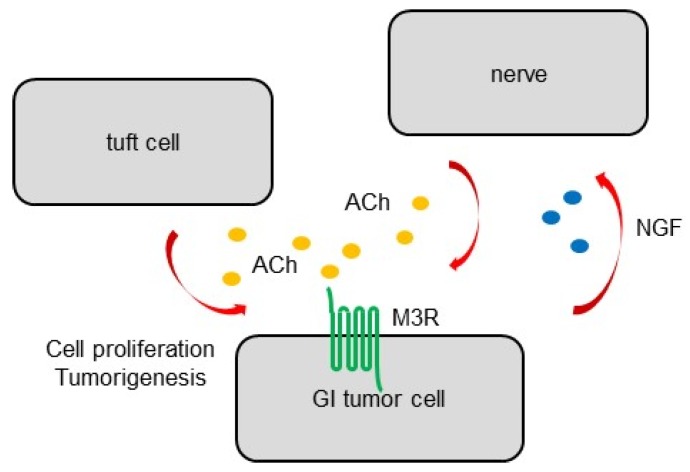
Schema of GI cancer cells and ACh sources. The nerve is the main source of ACh secretion. Dclk1+ tuft cells act as an alternative epithelial source of ACh. Nerves and tuft cells in the stomach and intestines expand at discrete times during carcinogenesis, leading to an increased ACh concentration in cancerous tissues. Cholinergic stimulation of the gastric epithelium induces nerve growth factor (NGF) expression, and, in turn, NGF overexpression in the gastric epithelium expands enteric nerves and promotes carcinogenesis.

Results of studies involving vagotomy have suggested that the vagus nerve is the main source of ACh secretion in the GI tract, but also that an alternative source of ACh exists in the GI epithelium. It has been reported that differentiated tuft cells, which commonly express doublecortin like kinase 1 (Dclk1), also express choline acetyltransferase (ChAT) and act as an epithelial source of ACh [48,63]. During carcinogenesis, tuft cells expand earlier and nerves expand later within cancerous tissues, resulting in the increase in ACh concentration in tissues. In particular, tuft cells expand dramatically in inflammation-associated cancer models and contribute to early cancer growth and the remodeling of the peritumoral neural microenvironment [48]. Furthermore, mouse models suggest that cholinergic stimulation of the gastric epithelium, in part, through ACh secretion from tuft cells, induces nerve growth factor (NGF) expression. NGF overexpression in the gastric epithelium, in turn, expands axonal growth from nerves in the lamina propria, and eventually promotes carcinogenesis by activating gastric stem cells [48]. Indeed, the ablation of Dclk1+ tuft cells and blockade of NGF/Trk signaling inhibit epithelial regeneration and tumorigenesis in an M3R-dependent manner, highlighting the importance of tuft cells as a component of the cancer niche. This feedforward ACh–NGF axis offers a compelling target for tumor treatment and prevention [48] (Figure 2). 

## 5. ACh Signaling and Colon Cancer

Evidence suggests that ACh-M3R signaling also plays a role in colonic carcinogenesis [64]. In *ApcMin* mice carrying the Apc gene mutation, the simultaneous knockout of the *Chrm3* gene leads to the reduction in intestinal tumor load. β-catenin nuclear staining is attenuated in *ApcMin*/+ *Chrm3*−/− mice compared to in *ApcMin*/+ *Chrm3*+/+ mice with tumors, suggesting a similar regulatory mechanism of Wnt activation by M3R signaling [65]. Another study demonstrated that M3R-deficient mice have an attenuated cell proliferation, tumor number, and tumor size in chemically induced colon tumor models [38]. In addition, treatment with bethanechol, a muscarinic agonist, increases colonic proliferation by upregulating selected matrix metalloproteinase (Mmp) genes and Wnt target genes such as Myc and cyclin D1 [66]. Collectively, these findings support important roles for M3R expression and activation in the progression of colon neoplasia [67]. M3R and its downstream signaling pathway may be a promising therapeutic target of colon cancer [68].

While the relationship between M3R and YAP signaling has been suggested in gastric cancers [48], it remains undetermined whether such a clear connection between these two pathways also exists in intestinal cancers. Several studies have suggested that YAP signaling generally promotes epithelial regeneration and tumor development in intestines, primarily by stimulating tissue stem cells and progenitors [56,69,70]. The YAP pathway also plays a role in the regulation of colonic cell death/apoptosis, invasion, or metastasis [71,72]. Nevertheless, given that the opposite effects by YAP signaling on intestinal tumor development have been reported [62,73], further studies would be required to understand the precise function of this pathway in nerve-dependent tumorigenesis.

During inflammation-associated colon cancer development, vagus nerves also mediate anti-inflammatory reflux in the spleen [74]. Vagus stimuli promote the secretion of trefoil factor 2 (TFF2), an anti-inflammatory peptide, from memory T cells, which leads to the suppression of myeloid cells and the alteration of the inflammatory microenvironment in colonic tumors. Myeloid cells are also known as a source of Wnt ligands in the intestines and contribute to epithelial regeneration [75]. Therefore, vagus nerve signaling and ACh signaling are involved in colonic carcinogenesis in a complicated manner.

As demonstrated in the stomach, Dclk1+ tuft cells contribute to colonic regeneration and homeostasis, at least, in part, through ACh/M3R signaling. The ablation of Dclk1+ tuft cells in the colon leads to a significant reduction in epithelial proliferation, and exaggerated epithelial damage, after dextran sodium sulfate (DSS)-induced colitis [76]. In contrast, the overexpression of NGF and subsequent increased nerve signaling promoted epithelial repair following colonic injury, whereas M3R knockout in the colonic epithelium worsened the outcome, in a DSS-induced colitis model [48]. Interestingly, the conditional knockdown of Apc in Dclk1+ tuft cells is insufficient to drive colonic carcinogenesis under normal conditions; however, DSS-induced colitis induces the development of poorly differentiated colonic adenocarcinoma in mice specifically lacking the Apc gene in Dclk1+ tuft cells [76], suggesting that Dclk1+ tuft cells also act as cancer-initiating cells under specific conditions. In addition, in vitro co-culture experiments showed that nerves fail to support organoid growth in the absence of Dclk1+ tuft cells, suggesting that Dclk1+ cells are involved in the integration of neuronal signals in the epithelium [76]. More recently, Goto et al. identified interleukin 17 receptor B (IL17RB) as a cell-specific marker of tuft cells, and showed that IL17RB regulates tuft cell-derived cancer stem cell function and could be a therapeutic target [77].

## 6. Conclusions

M3R signaling regulates cell proliferation, survival, and tumorigenesis by activating various signaling pathways, such as MAPK, Akt, YAP, Wnt, and NGF, in GI cancers. In addition to nerves, tuft cells serve as sources of ACh in the GI tract and contribute to regeneration and homeostasis. Components and involved signaling pathways in nerve–tumor interactions may be a promising target for the treatment of cancers of the GI tract.
